# Microfluidic neurite guidance to study structure-function relationships in topologically-complex population-based neural networks

**DOI:** 10.1038/srep28384

**Published:** 2016-06-22

**Authors:** Thibault Honegger, Moritz I. Thielen, Soheil Feizi, Neville E. Sanjana, Joel Voldman

**Affiliations:** 1 Department of Electrical Engineering and Computer Science, Massachusetts Institute of Technology, Cambridge, MA 0213, USA; 2 Univ. Grenoble Alpes, LTM, F-38000 Grenoble, France; 3 CNRS, LTM, F-38000 Grenoble, France; 4 Computer Science and Artificial Intelligence Laboratory, Massachusetts Institute of Technology, Cambridge, MA 02139, USA; 5 Broad Institute of MIT and Harvard, Cambridge, MA 02142, USA; 6 McGovern Institute for Brain Research, Department of Brain and Cognitive Sciences, Department of Biological Engineering, Massachusetts Institute of Technology, Cambridge, MA 02139, USA

## Abstract

The central nervous system is a dense, layered, 3D interconnected network of populations of neurons, and thus recapitulating that complexity for *in vitro* CNS models requires methods that can create defined topologically-complex neuronal networks. Several three-dimensional patterning approaches have been developed but none have demonstrated the ability to control the connections between populations of neurons. Here we report a method using AC electrokinetic forces that can guide, accelerate, slow down and push up neurites in un-modified collagen scaffolds. We present a means to create *in vitro* neural networks of arbitrary complexity by using such forces to create 3D intersections of primary neuronal populations that are plated in a 2D plane. We report for the first time *in vitro* basic brain motifs that have been previously observed *in vivo* and show that their functional network is highly decorrelated to their structure. This platform can provide building blocks to reproduce *in vitro* the complexity of neural circuits and provide a minimalistic environment to study the structure-function relationship of the brain circuitry.

Network analysis is growing as an approach to model the complexity of the human brain. Sporns *et al*.^1^ have shown that among the connectomes of primates, regular and small (3, 4 or 5 nodes) structural motifs form characteristic network building blocks that, when assembled computationally, resemble real brain networks, including small-world attributes. Reproducing *in vitro* very simplified models of *in vivo* increased complexity networks will help understanding of the structure-function relationship and *in fine* serve as minimalistic reproductions of part of the human connectome, which could then be used to create high-throughput synthetic models of neurodegenerative diseases^2^, high-throughput assays for drug discovery when coupled with physiologically relevant cells^3^, and to create biological neuronal computers on-chip^4^.

Several studies have shown that *in vitro* neural networks share the same network characteristics as their *in vivo* counterparts^5^,^6^. The main constraint to reproduce a complex network *in vitro* is the ability to create a complex graph, since *in vivo* networks are represented structurally by non-planar graphs. *In vitro* two-dimensional systems are inherently unable to recapitulate the topological complexity of *in vivo* networks^7^, and only a maximum of three populations (n > 100) have been connected so far^7^,^8^. Several three-dimensional patterning approaches have been developed (chemical cues^9^, colloidal support^10^,^11^ or building blocks^12^) but none have demonstrated the ability to control the level of connectivity between populations of neurons that is required to access the non-planar network complexity of *in vivo* networks. Moreover, existing 3D systems are unable to observe inter-population activity of individual neurons by conventional means because of their inability to provide wide field of view visual access to all neurons at the same time.

We have previously shown that AC electrokinetic forces associated with geometrical constraints can repel neurite growth in a plane^13^. Here we present, for the first time, a method to create *in vitro* neural networks of arbitrary complexity by using such forces to guide, accelerate, slow down and push up neurites in un-modified collagen scaffolds, allowing 3D intersections of selected neuronal populations that are plated in a 2D plane. Collagen is one of the main components used in tissue engineering^14^ and remains the gold standard for producing physiologically relevant *in vitro* scaffolds. We used a combination of compartmentalized microchannels to isolate populations of rat hippocampal neurons and microelectrodes to guide neurites. Each population was independently plated and allowed to project to the other ones within microchannels selectively filled with collagen, which acts as a 3D neurite scaffold. We first demonstrated that when applying an AC field within the collagen scaffold using coplanar electrodes on the channel bottom, neurite growth can be controlled accordingly to the applied field. Further, we developed a method to create tunable neurite crossings to allow the creation of complex non-planar networks. We demonstrate that this technique can be used to reproduce structurally *in vitro* human brain basic motifs, a term referred by Sporns^1^, by combining population-based neural networks, neurite diodes, and neurite bridges. Finally, we analyzed the intra and inter population functional activity of the topologically structured network over time and demonstrated the emergence of small-world organization within those motifs.

## Results and Discussion

### Selective patterning of collagen scaffolds in a compartmentalized electro-microfluidic chip

**Figure 1:**
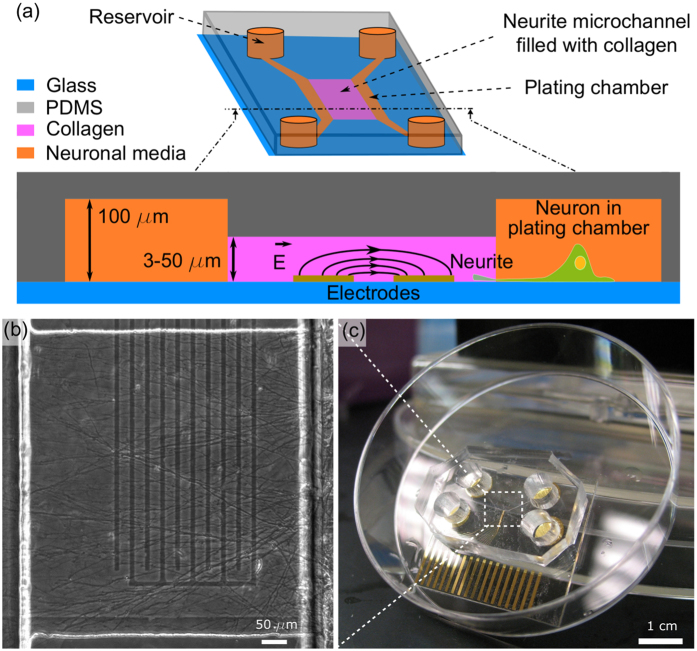
Overview of the electro-microfluidic compartmentalized chip. (**a**) Schematic of the chip that comprises open reservoirs where neurons are injected and flowed through the plating chambers. Neurites can grow into selectively filled collagen scaffolds of varying heights. (**b**) Bright field image of developing neurites in a 5 μm height scaffold without any field after 6 days *in vitro* (DIV). Neurites showed oriented growth from the cell body compartment within the collagen scaffold. (**c**) Picture of the microfluidic chip in a petri dish. The electrical connections are visible on the lower part of the chip.

To provide a 3D scaffold for the neurites, we selectively patterned collagen inside a double-layer microfluidic channel without the need of extra patterning channels^15^ or additional equipment^16^. For shallow channels, our method is based on capillary flow balancing to perfuse defined areas of the microfluidic chip with collagen, followed by removal of collagen from unwanted areas with acetic acid (Fig. S1). This results in deep open channels suitable for plating cells bodies along with shallow scaffold-laden channels for the growing neurites (Fig. 1).

### AC fields can guide, accelerate, slow down and push up neurites growths in collagen scaffolds

Incorporating co-planar electrodes into the microfluidic chip, we first assessed if AC electrokinetic forces and microchannel geometrical constraints can direct neurite growth in three dimensions in unmodified collagen scaffolds. Our previous description of the AC electrokinetic effect^13^ on developing neurites shows that individual growth cones are repelled from the electrode edges at high frequencies and are not harmed by the AC electrical current; neurites continuously grow on top of activated electrodes, and the growth cones maintain their ability to form functional synapses. More specifically, we have shown that the repelling of the growth cone is not due to electrolysis or heating and unlikely due to actin filament alignment. Within a certain voltage and frequency ranges that we use in the present work, the repelling effect is consistent with AC electrokinetic forces.

**Figure 2:**
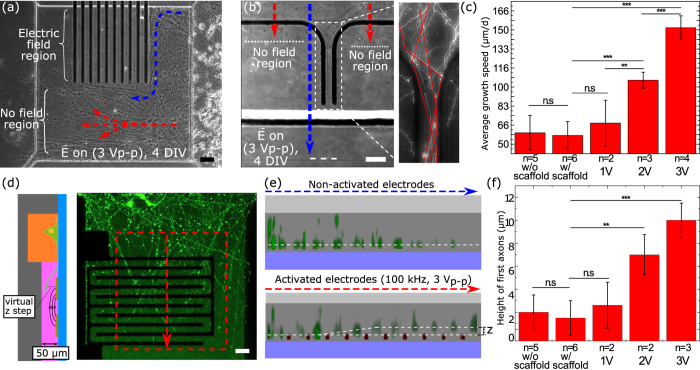
Neurite repelling in 3D gels. (**a**) Image of neurite growth in a 5 μm-thick collagen-filled channel after 4 DIV with activated electrodes (150 kHz, 3 Vpp). **Speeding up**. (**b**) Picture of neurite development in a 5 μm high collagen-filled microchannel between electrodes in a funnel-like design after 4 DIV and activated electrodes (100 kHz, 3 Vpp). The inset shows a fluorescent staining of the final growth on which the trajectories of neurites have been highlighted in red lines. (**c**) Average neurite growth speed after 6 DIV under varying applied voltage and scaffold conditions. The *n* indicates the number of independent experiments repeats. **Pushing neurites up in 3D.** (**d**) Schematic and image of neurites growing in a 50 μm-thick collagen-filled channel after 6 DIV (100 kHz, 3 Vpp). (**e**) Side-view confocal images of neurites traversing the channel along the red line for inactivated (top) and activated (bottom) electrodes. (**f**) Z-directed neurites deflection above activated electrodes with varying applied voltage and scaffold conditions. The *n* indicates the number of independent experiments repeats. All scale bars indicate 50 μm.

First, when the channel height is <5 μm, we found that when activated, the electrodes applied AC electrokinetic repelling forces that the neurites in gels cannot bypass (Fig. 2a and Fig. S2), likely due to both the channel’s steric constraints and the repelling effect. The effect was voltage-dependent, with voltages above 2 V and below 4 V, repelling neurites without injury, i.e. the neurites continued to grow after turning off the field (Fig. S2). This thus provides a contactless, electrode-patterned and dynamic way to guide neurites in a plane within a window of operation, where 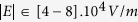.

Second, by orienting the electrodes parallel to the neurites (i.e., funnel shape), we find that the average growth speed of the neurites increased (Fig. 2b,c) more than twice compared to pure collagen, which may be the result of the spatial confinement of the cone probing area and, to our knowledge, is the first demonstration of a non-chemical and contactless approach to boost multiple neurites’ growth speed^17^,^18^ in a plane and collectively. As shown on the inset in Fig. 2b, the trajectories of the neurite suggest that the growth cone bounces upon approaching the activated electrodes, which results in a final linear trajectory parallel to the electrode direction. Although other optical approaches^19^ have shown the ability to focalize actin filaments inside the developing growth cone, resulting in guiding of individual growing neurites, our approach results in a control of multiple developing growth cones.

Third, by increasing the channel height to ~10 μm, we significantly slowed down the average neurite growth speed as compared to growth extension on glass or in bare collagen (Fig. S3). In this case, it is possible that the growth cone is under a competition between the physical confinement of the microchannel and the electric field repelling effect. At this height, collagen fibers are aligned parallel to the groove due to the flow-filling method and provide “fast” tracks for the growth cone, as recently shown in matrigel scaffolds^20^, which is consistent with the enhanced speed observed without the field activated.

Finally, when the channel height is ~50 μm, we can push up neurites (in *z*) and away from the electrodes (Fig. 2d,e and Fig. S5), creating a region near the electrode surface where the neurites are not able to enter and whose height increases with the electric field strength (Fig. 2f, Fig. S4). Beyond the electrode, we observe that some neurites grow back down to the lower plane, near the electrodes, suggesting that neurites close the repelling zone of the electrodes can go back down once they are past the repelling region. In thick scaffolds, higher field strengths are possible without injuring the growth cones because the neurites are not in direct contact with the electrodes. Although application of AC fields to pattern cells in scaffolds has been demonstrated previously^21^, here we demonstrate for the first time that AC fields can extend throughout the collagen scaffold to control the orientation of neurites directly within the scaffold itself. Because of the spatially localized nature of the AC field gradient effect on the growth cone growth, the limit in the z-direction in the depth of the scaffold deflection is ~10 μm from the planar electrodes. To fully control the growth of neurites in the entire depth of the scaffold, 3D electrodes embedded in the scaffold would be necessary.

### Neurite bridges

**Figure 3:**
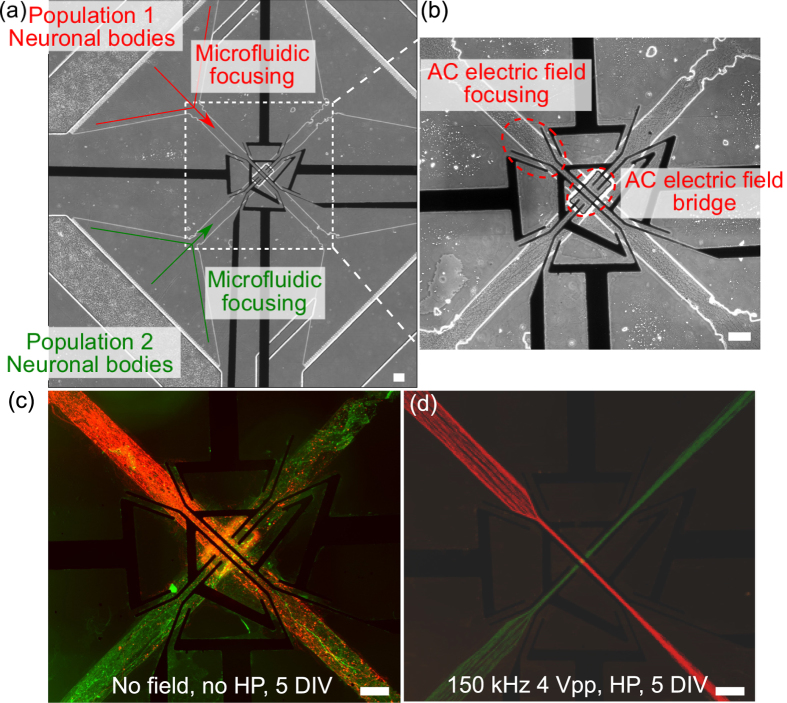
Neurite bridges. (**a**) Stitched transmission picture of the neurite bridge device. Neuronal bodies were placed in fluidly isolated plating chambers and individually stained. Compartmentalized grooves allow neurites to develop and focus towards the bridge region filled with collagen. (**b**) Close-up image of the bridge area composed of funnel electrodes that will focus the neurite beam before contactless pushing up in the collagen scaffold. (**c,d**) Fluorescence picture of the bridge region (**c**) without and (**d**) with the field activated. Additionally, a hydrostatic pressure gradient was applied from top-left to lower-right and lower-left to top-right to boost neurite growth. All scale bars indicate 100 μm.

To illustrate how these unit operations can be combined for creating neural networks, we developed a method to create tunable neurite crossings. We combine the stopping, funnel, and pushing functionalities to create a “neurite bridge”. Neurites projected by two populations of E18 hippocampal rat neurons, labeled with either EGFP and tdTomato, are focalized by geometrical constraints (microfluidic channel constrictions) and by AC electrokinetic forces (funnel shape electrodes) (Fig. 3a,b). At the intersection, a collagen extrusion (50 μm thick) is selectively injected in the microchannels so that the neurite beam, that encounters electrodes perpendicular to their path, can be pushed up in the scaffold. Without any field, neurites sprout in all channels (Fig. 3c), with 78 ± 12% (n = 3) of neurites growing in the same direction as the initial channel. Neurons from both populations were connected via the central region but also projected neurites in all channels.

On the contrary, when the field was activated, the neurites were focused into a beam of neurites, allowing them to be pushed up and bridge without sprouting in the other channels (Fig. 3d) with a directional growth of 98 ± 2% (n = 3) in the same direction. We observed a high degree of fasciculation of neurites at the exit of the bridge. Alternatively, a bidirectional connection can be created between two facing populations (Fig. S5). To boost neurite growth in the channel direction, it was found that maintaining a hydrostatic pressure (HP) difference between two facing plating chamber increased neurite growth by at least 30% (it took 8 to 9 DIV for neurites to connect with the other compartment without HP versus 5 to 6 DIV with HP).

### *In vitro* structural motifs

**Figure 4:**
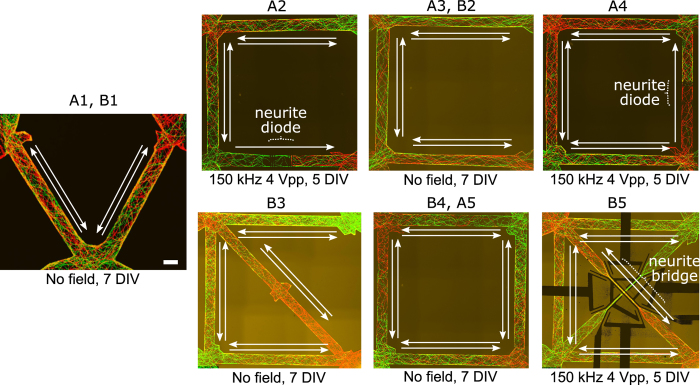
*In vitro* human brain basic motifs. Fluorescence pictures of motifs. Directionality of the structural connection is indicated with white arrows. Motifs with three nodes present bidirectional connections (A1, B1) whereas the ones with four nodes can have directional (A2; A4), bidirectional (A3, B2; A5, B4; B3) or intersecting (B5) connections. Directionality of neurites from one population to another was imposed by neurite diodes and intersections by neurite bridges. Scale bar indicates 100 μm.

Motifs are small network building blocks that, when assembled together, describe the structure of a complex system. They can be distinguished according to the size of the motif, equal to the number of nodes and the number of pattern of interconnections^22^. Sporns *et al*. have observed that structural motifs are consistently encountered in the cortical connection matrices of macaque, cat and *C. elegans*^1^. Those motifs may also be the basic motifs of the human brain connectome^23^, although this has not yet been directly observed. Using the AC electrokinetic confinement of neurites that we present here along with neurite “diodes” that enable one-way connections between two neuronal populations^13^, we have reproduced the topology of such motifs *in vitro* (Fig. 4), namely basic brain motifs as suggested by Sporns. Motifs composed of three nodes present bidirectional connections whereas the ones with four nodes can have unidirectional (A2, A4), bidirectional (A3, A5, B3) or intersecting (B5) connections. Our motifs were named according to the motifs highlighted by Sporns. The structure of such basic brain motifs can be reproduced *in vitro* for the first time by combining population-based neural networks, neurite diodes^13^, and neurite bridges.

### Structure-function relationship in basic brain motifs

**Figure 5:**
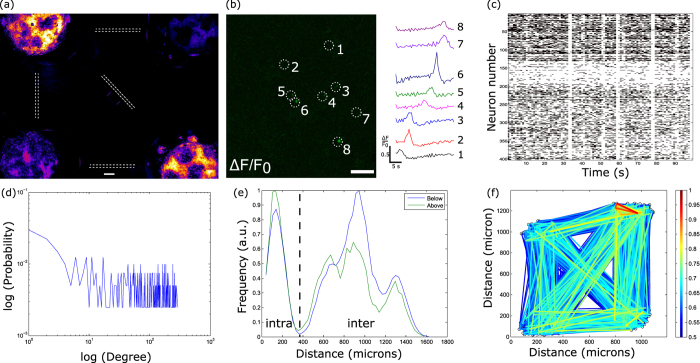
Functional structure of B5 motifs. (**a**) Fluorescent calcium imaging of B5 where superimposed arrows show the structural organization of the network. Scale bar indicate 100 μm. (**b**) Picture of a ΔF/F0 image of a node where identified spiking neurons are highlighted with white dashed circles and matching temporal plot. Scale bar indicates 50 μm. (**c**) Temporal plot of the entire network’s spikes after thresholding. (**d**) Plot of the degree distribution of the network. (**e**) Plot of the distribution of the network-level correlation coefficients as a function of distance. (**f**) Plot the functional network of B5 where each identified neuron was spatially placed according to its structural coordinates.

To assess the structure-function relationship of these motifs, we observed the spontaneous activity of each motif at the network level via calcium imaging after full maturation *in vitro*^5^ (21 DIV, Fig. 5a). Because all neuronal bodies are plated on a surface, the spontaneous activity of individual neurons within each population (or each node) was observed and analyzed to perform intra and inter population cross-correlation analysis. Neurons with detectable calcium signals were numbered (n = 400, Fig. 5b). Spontaneous spikes were detected, which showed that the nodes were mature enough to generate inter node communications at the network level (Fig. 5c). The degree distribution (Fig. 5d) is decreasing with increasing degree, similar to a decreasing power law, which is consistent with a scale-free network structure^24^. The extracted network parameters yield a characterization of such networks as small-world networks with a scale-free topology (Clustering coefficient σ = 5.24 ± 0. 42, shortest path length λ = 1.07 ± 0.041, small-world parameter σ/λ = 4.87 ± 0.098, for the B5 motif (n = 4), all parameters for each motif are shown in Table T1). The distance distribution of the correlation coefficients shows that cells close to each other (intra population) were strongly correlated, but that there also exist long-distance correlations (inter population) (Fig. 5e). The resulting functional network structure (Fig. 5f) reveals an unexpected connectivity pattern not only for the B5 motif but for all motifs (Fig. S7). Although all motifs presented both highly correlated intra and inter population communications with small-world properties, all nodes were fully functionally connected to the others independent of their topological connections (Fig. 4). Such results suggest that *in vivo* neural functional motifs are not necessarily supported by the underlying structural network and that uni- or bi-directional connections between directly connected nodes have a significant impact on the global network connectivity.

The ability to reproduce *in vitro* minimal circuits that mimic aspects of brain connectivity is appealing because it would open access to fundamental experiments on the structure-function relationship^25^ but also for therapeutic screening of the functional connectivity of an injured brain^26^. In contrast with other methods, our approach makes it possible to recreate minimalistic population-based networks by focusing on their connectivity, in the same way that the human brain is described as a complex network. Such description of the brain is based on the extraction of nodes and connections observed by imaging data (structure and/or function) of brains and leads to the generation of adjacency matrices^22^,^25^. These matrices are then used to compute connectomes by integrating both its structural and functional connectivity. By using the reverse process, *in vitro* neural networks can be represented by adjacency matrixes that describe structural connections between populations of neurons. Translating those matrixes into compartmentalized microfluidic chip designs results in a set of reservoirs in which the populations will be plated and connected by selectively-filled collagen microchannels with patterned electrodes. As an analogy with their *in vivo* counterpart, those *in vitro* networks are based on populations of neurons that may represent certain parts of the brains and the connecting microchannels, their structural connections. By using AC electrokinetic forces to structure in 3D the connections of neuronal populations that are plated in a 2D plane, *in vitro* neural networks of any complexity can be formed. As an example, the adjacency matrices of the neurite bridges are given by C_1_ (not activated, Fig. 3b) and C_2_ (activated, Fig. 3c):


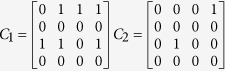


The ability of neurite populations to cross without connection enables the formation of complex population-based neural networks. Such networks are non-planar by essence, according to graph theory. A planar graph is a graph that can be embedded in the plane, i.e., that can be drawn on the plane in such a way that its edges intersect only at their endpoints. Hence, a non-planar graph cannot be drawn in such a way that no edges cross each other. In practice, this means that non-planar graphs of neurons require three-dimensional crossings. A theoretical proof shows that, by combining the unit operations presented above, a neural network of arbitrary complexity can be created by devolving intersections of more than two populations of neurites into several intersections of two populations (Supplemental Note 1).

Our work provides a number of tools for creating minimalistic *in vitro* neural networks. First, it suggests segregating populations of neurons in nodes. It is thus possible to co-culture more than two^8^,^27^ or three^28^,^29^ types of neurons in different reservoirs and to connect them together in the same chip by optimizing the relative positioning of nodes without the connectivity constraints. The number of plated cells in each reservoir can be tuned by the plating density and can contain up to thousands of neurons per node. Such approach also allows for recording/stimulating the activity of the entire population with single cell resolution. Contrary to neurospheroid-based approaches^30^,^31^, access to all the somas of the population in a single plane can offer the capacity to couple our method with neural activity readers (calcium activity monitoring used in this work, microelectrode arrays or light-sheet functional imaging^32^) or activators (optogenetics with modified channelrhodopsin neurons and a focalized in-plane stimulation beam). Second, it combines both geometrical and AC field growth constraints to control the connections between the nodes, allowing constraint-free positioning of nodes relatively to each other. Compared to existing neurite guidance techniques, either by controlling adhesive cues^33^,^34^,^35^ or using microfluidic grooves^36^ or both^37^ or other techniques^38^,^39^, our method allows a dynamic multiplexed manipulation of neurites within the same groove, which enables the precise relative positioning of several neurites at the same time. Using our technique, *in-vitro* neural networks would not be limited by their structural connectivity between nodes, allowing the creation of complex networks with more than three nodes.

## Conclusion

We have shown that this platform can be used as a minimalistic environment to help studying the structure-function relationship that is currently explored in the inherently highly complex human brain. Not surprisingly, the functional connectivity of neural circuits is more complex than supported by a single translation of to a direct matching structure. However, current *in vivo* imaging techniques reveal basic functional patterns that should take place within the entire connectome. It seems that the global inter population activity is mainly dictated by the intra population connectivity where nodes and hubs play a significant role both in redundancy of the transmitted signal and also in reconfiguration when nodes are affected by degenerative diseases^40^. We suggest that further work should focus on analyzing the different neuronal types within a population and monitoring the impact of the entire network. Whereas researchers have been focused on recreating adhesion motifs for creating *in vitro* models of the brain, this work suggests that the main issue relates to connecting populations with physiologically relevant connectivity patterns. Those physiologically relevant minimalistic connectomes-on-a-chip could help understand the high complexity of true brain circuitry.

## Materials and Methods

### Microfabrication of electrokinetic devices

The microfluidic chips were fabricated on standard microscope glass slides (25 × 75 × 1.1 mm and 20 mm × 60 mm × 170 μm for the 50 μm height channels, Sigma). A 10/100 nm Ti/Au bi-layer was deposited on the slides using e-beam deposition. The layer was then structured using standard lithography and wet-etched for 4 min in 250 mL H_2_O + 200 mL HCl + 100 mL HNO_2_. Subsequently, a second wet-etch of 10 min in HCL:Water (2:1) was performed to remove remaining titanium.

The microchannels comprise two types of components: high channels (100 μm) for cell injection and shallow channels (5–50 μm in height) for neurite growth. They were molded from a SU-8 (Microchem) master that was fabricated with a two-step lithography process with thin (SU-8 2007) thick (SU8-2050) resists. Microchannels were then molded with degassed and cured PDMS (9:1 mass ratio with curing agent, Sylgard 184, Dow Corning). The microgrooves where manually aligned under a binocular after air plasma exposure (2 minutes) and immersion in methanol (5 minutes)^41^,^42^. The assembled chip was cured at 100 °C for 30 minutes. After the initial mold, a plastic master was fabricated for further replication of the device^43^. The PDMS molds were then manually aligned to the electrodes using a stereo microscope (M80, Leica) and bonded on the glass substrate after 2 minutes exposure to air plasma. The assembled chip was cured at 80 °C for 30 min.

Several microfluidic chips were constructed in this way. To evaluate the effect of AC fields on neurite growth in collagen scaffolds, two-compartment chips were made of rectangular microchannels (length: 4000 μm; width: 500 μm; height: 100 μm) separated by arrays of planes (length: 600 μm; width: 600 μm; height: 5 to 50 μm). The neurite bridge chip was a four compartments chip made of 5-mm punched inlet and outlet reservoirs connected to four inner reservoirs (length: 1000 μm, height: 100 μm) placed in an square. Each reservoir was connected to the other through microchannels (length: 500 μm; width: 50 μm; height: 5 μm).

### Surface treatment and coating

To clean the glass substrates (with electrodes), they were boiled for 1 h in 7X detergent (MP Biomedicals), rinsed for 10 s in DI water, cleaned with Aceton, Isopropanol and DI-water and finally baked for 2 h at 200 °C in an oven. After baking, the substrate was plasma-cleaned and bonded to the PDMS microfluidics. Channels were filled with Poly-D-Lysine (0.1 mg/ml, Sigma) and incubated at 37 °C for at least 20 h. To remove loose PDL, channels were washed twice with DI water without emptying the main channel. Subsequently, the channels are filled with Laminin (20 μg/ml, Sigma) and incubated at 37 °C for 2 h. Laminin was aspirated and the channels washed tree times with Plating Media (DMEM + 10% FBS + 1% PS + 1% L-Glutamin) from one side to the other. A chip functionalized in this fashion was stored for max. 2 h prior to cell seeding at 37 °C. Neurobasal-B27 containing 2 mM glutamine and 100 U/ml penicillin/streptomycin (hippocampal culture medium).

### Selective patterning of collagen scaffolds

Collagen (10 mg/ml, Gibco) was mixed on ice with a buffer solution (250 mM HEPES in 2X PBS, pH 7.4 with 2 M NaOH) for 1 min. The ratio of collagen to buffer depended on the desired final collagen concentration but was close to 1:1. Before pipetting into the chip, the collagen solution was incubated 10–30 min on ice to control the fiber thickness as proposed in ref 44. First, the bonded chip was functionalized with poly-d-lysine and laminine to allow adhesion of the neuron soma in the cell reservoir. Second, the complete microfluidic channel was filled with collagen type I solution through the scaffold inlet and incubated at 37 °C until complete gelation. Following, acetic acid (AA) (0.2 M, pH 3.5) was pipetted into the cell reservoir to destabilize the collagen. AA is known to disrupt the stabilizing hydrogen bonds between collagen fibrils, resulting in solubilisation of the collagen scaffold^45^. To prevent acid from etching into the microfluidic channel, a hydrostatic pressure was applied by pipetting cell media into the inlet. After 30 min etching at 37 °C, the destabilized scaffold was carefully aspirated through the cell outlet. To remove acid and residues, the channel was flushed three times with buffered cell media. Fig. S1 presents an illustration of both capillary and etching methods.

### Dissection and cell culture

All animal work was approved by the MIT Committee of Animal Care and Division of Comparative Medicine, and abided by institutional, state, and federal guidelines for animal welfare. Hippocampi were harvested from E18 Sprague Dawley rats (Charles River Laboratories,), and digested in ice-cold Hank’s balanced salt solution (HBSS), buffered with 10 mM HEPES, pH 7.3. The tissue was digested by a 30 min incubation in 2 ml of HEPES buffered HBSS containing 20 U/ml of papain (Worthington Biochem.), 1 mM EDTA and 1 mM L-cysteine. Next, the tissue was rinsed three times with 8 ml of hippocampal culture medium. The cells were gently triturated in 1 ml of hippocampal culture medium, counted with a hemocytometer, and plated at a density of 35,000 cells/mm^2^. The cells were maintained at 37 °C, 5% CO_2_. The cell medium was renewed for 50% every 3 days. To maintain hydrostatic pressure, the reservoirs of the neurons compartment were systematically filled with media every day and the others were emptied without drying the reservoir surface.

### Neuron seeding in device

Before seeding, the reservoir of the microfluidic chip was emptied without removing the media from the microchannel. For each inlet/outlet reservoirs, 6 μL of plating media was placed in the outlet and immediately after, 4 μL of high density (>8 10^6^ cells/mL) harvested neuron solution was placed at the inlet reservoir. The chip was returned to the incubator for 5 minutes in order to let the neurons adhere on the coated glass and the seeding process was repeated 3 times. At the end, the input and output reservoir were quickly filled with hippocampal culture medium and chips were returned to the incubator and plugged into the *in vitro* platform to apply AC fields that was described elsewhere^13^. For the basic brain motifs, the AC field was applied until successful growth completion, at which point the field was turned off. Validation was effective once neurites passed the critical point: for the bridge device, once neurites extended over the 50 μm scaffold, and for the diode, after activation of the final electrodes. Such validations were device dependent when mixing bridges and axons. Devices were then put back in culture and maintained until functional observation.

### Lentiviral cloning and transduction

Each population of neurons was transduced independently with a fluorescent lentivirus. Because of the fluidic isolation between cell body compartments and the lifetime of the virus, it was possible to stain both populations of neurons after 4 DIV with 4 μL of virus diluted in cell culture medium.

For each fluorescent lentivirus, either tdTomato or EGFP was cloned after the CMV promoter and before a Woodchuck Hepatitis Posttranscriptional Regulatory Element (WPRE) in a lentiviral transfer plasmid and amplified in Stbl3 cells. To produce the viruses, 3 million HEK293FT cells (Life Technologies) at low passage (less than 10) were seeded the day before transfection in a T-225 flask in DMEM supplemented with 10% FBS (Hyclone). Cells were transfected in OptiMEM using 100 μl of Lipofectamine 2000 and 200 μl of Plus reagent (Life Technologies) with 20 μg of the transfer plasmid (either tdTomato or EGFP), 15 μg of psPAX2, and 10 μg of pVSVg (Addgene). After 6 hours, the media was removed and replaced with DMEM supplemented with 10% FBS and 1% BSA. After 60 hours, the supernatant was removed and centrifuged at 3000 rpm for 10 minutes at 4 °C. This supernatant was filtered through a 0.45 μm low protein binding filter (Millipore). To achieve 300X concentration of viral particles, the filtered lentivirus was ultracentrifuged (Sorvall) at 24,000 rpm for 2 hours at 4 °C and then resuspended overnight at 4 °C in D10 supplemented with 1% BSA. Aliquots were stored at −80 °C until neuron transduction.

### Image acquisition

Images were acquired with an Axiovert 200 M (Zeiss) fitted with a cooled CCD camera LaVision ImagerQE (LaVision) and an automated stage Ludl MAC 5000 (Ludl). The microscope was controlled with Metamorph software (Molecular Devices) and images were analyzed using ImageJ and Matlab (The Mathworks) software. Sets of images were stitched together with ImageJ plugin for Fiji^46^.

### Image analysis to quantify the number of neurons

Neurons body were counted on stitched images with ImageJ plugin MOSAIC^47^.

### Image analysis to quantify deviation angle, growth extension and height of neurites

The quantification of neurites alignment and length was performed as follow: images of n samples containing one channel with collagen scaffold were analyzed for each condition and quantified using Matlab (MathWorks). One sample contained a minimum of 50 neurites. A threshold value of the reflection intensity was defined to isolate neurite from background. For the deflection angle, by fitting an ellipse to the major axis of each neurite, the angle of the neurite to the main channel direction was determined. The orientation of neurites parallel to the alignment direction corresponded to an angle of 0°. The angular distribution of neurite was determined based on the relative frequency of orientation angles (classified into bins of 180 angles) and by a fit to a Gaussian full width at half maximum (FWHM). Deviation of the initial trajectory was defined such as the angle at the evaluation point along the neurite has more than 10% change compared to the one 50 μm (5 times the growth cone diameter) before. Deviation is then recorded to be the relative difference between those to angles. For the average growth speed, the origin of the neurite was taken at the beginning of the microchannel. The end of the neurite was manually tracked until a visible growth cone was pinpointed using ImageJ Simple neurite tracer^48^ or when possible NeuriteTracer^49^. Changes in direction along its course were taken into account by tracing along the entire length of each extension. Extensions that were solitary and clearly isolated were measured only to exclude the possibility of mix-up with other extensions. The average growth speed was calculated by dividing the measured extension length by the number of DIV reported on each figure.

Neurite height in 3-D was determined by post-processing of fluorescent confocal image stacks in Matlab. The script contained a Gaussian filter to reduce noise and to determine the center of the neurite. The plot contains the detected centers in the individual slices of the channel.

### Statistical analysis

For neurite stoppage analysis, differences were addressed by an unpaired Student’s t-test from two independent experiments in which each experimental condition was performed in duplicate. For all analysis: *p-value < 0.05; **p-value < 0.01; ***p-value < 0.001.

### Calcium imaging and network analysis

The neurons were stained with Oregon green BAPTA1 (Life Technologies) for 1 hour and washed three times with the medium. The entire network was observed for more than 10 minutes on a Nikon microscope with spinning disc fluorescence. Acquisition time was 500 ms every 500 ms (2 Hz sampling), which is sufficient to determine functional architecture^11^,^50^,^51^,^52^,^53^. The images were then parsed with the Matlab code provided by^50^ where network structures are analyzed by applying cross-covariance signal processing and graph theory to single-cell recordings, according to cross-covariance analysis^54^ and graph theory applied to small world networks^55^. Neurons were first segregated by size (10 μm) over the entire experiment duration. For all localizations, Δf/f_0_ was monitored. Based on each spontaneous spike, a global threshold (where for each pixel, Δf/f_0_ was larger than the average pixel value for each image) was applied to all four populations and the inter-population activity was plotted over time with a randomized number so that the cross correlation process would not be biased by proximity effects. The inter population correlation matrix was extracted, which allowed computation of the adjacency matrix for each motif and the correlation coefficient spatial distribution (Fig. S8).

## Additional Information

**How to cite this article**: Honegger, T. *et al*. Microfluidic neurite guidance to study structure-function relationships in topologically-complex population-based neural networks. *Sci. Rep.* **6**, 28384; doi: 10.1038/srep28384 (2016).

## Supplementary Material

Supplementary Information

Supplementary Figure S5
