# Supplementary material for: Microfluidic neurite guidance to study structure-function relationships in topologically-complex population-based neural networks
Source: Sci Rep. 2016 Jun 22;6:28384. doi: 10.1038/srep28384 (PMC4916598; doi:10.1038/srep28384)
Supplement: Supplementary Information [file srep28384-s1.doc]

Microfluidic axonal guidance to study structure-function relationships in topologically-complex population-based neural networks

Thibault Honegger, Moritz I. Thielen, Soheil Feizi, Neville E. Sanjana and Joel Voldman

Supplementary information


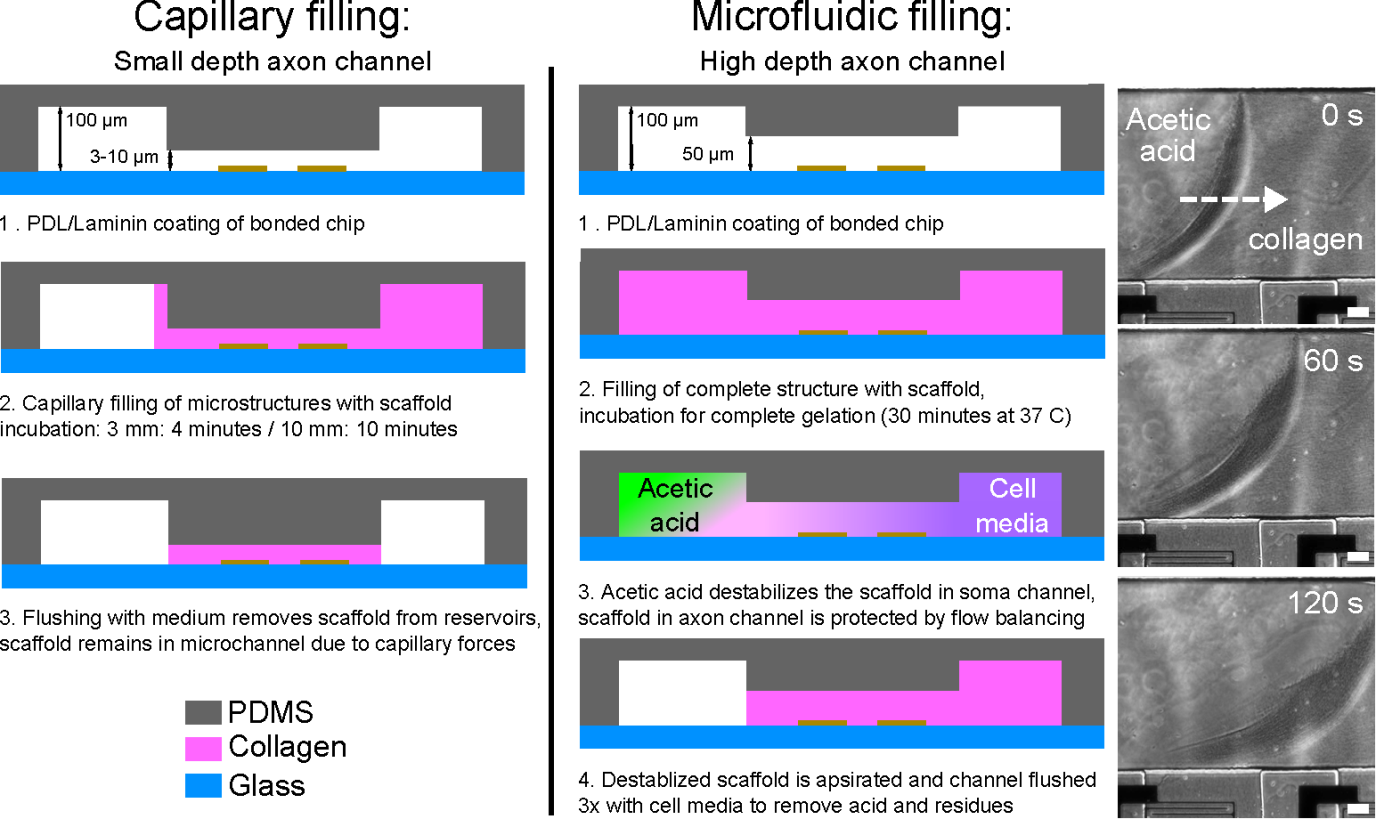


Figure S1: **Fabrication of compartmentalized chip selectively filled with collagen**. Illustrations of the protocol using capillary filling and microfluidic patterning. For capillary filling, (1) shows the electro-microfluidic chip sealed with functionalized plating chambers and electrodes aligned with the neurite microchannels. (2) Collagen is filled on ice from one plating chamber, and removed directly by filling the chambers with media. (3) After gelation, the microchambers are filled with collagen. For microfluidic filling, (1) shows the electro-microfluidic chip sealed with functionalized plating chambers and electrodes aligned with the high aspect ratio neurite microchannels. (2) After filling and complete gelation of the structure with collagen, the acid etches the cell reservoir while the microchannels are protected by application of a hydrostatic pressure from the scaffold filled reservoir (3). After the destabilized scaffold is aspirated (4), excessive acid and remainders are removed with buffer solution. From the time-lapse images an etch rate of ~500 μm/min can be derived after destabilization of the collagen through the AA. As a result, scaffold remains only in the electrode channel and in the media reservoir while the cell reservoir is empty and ready for the seeding of cells. Scale bar indicates 100 µm.


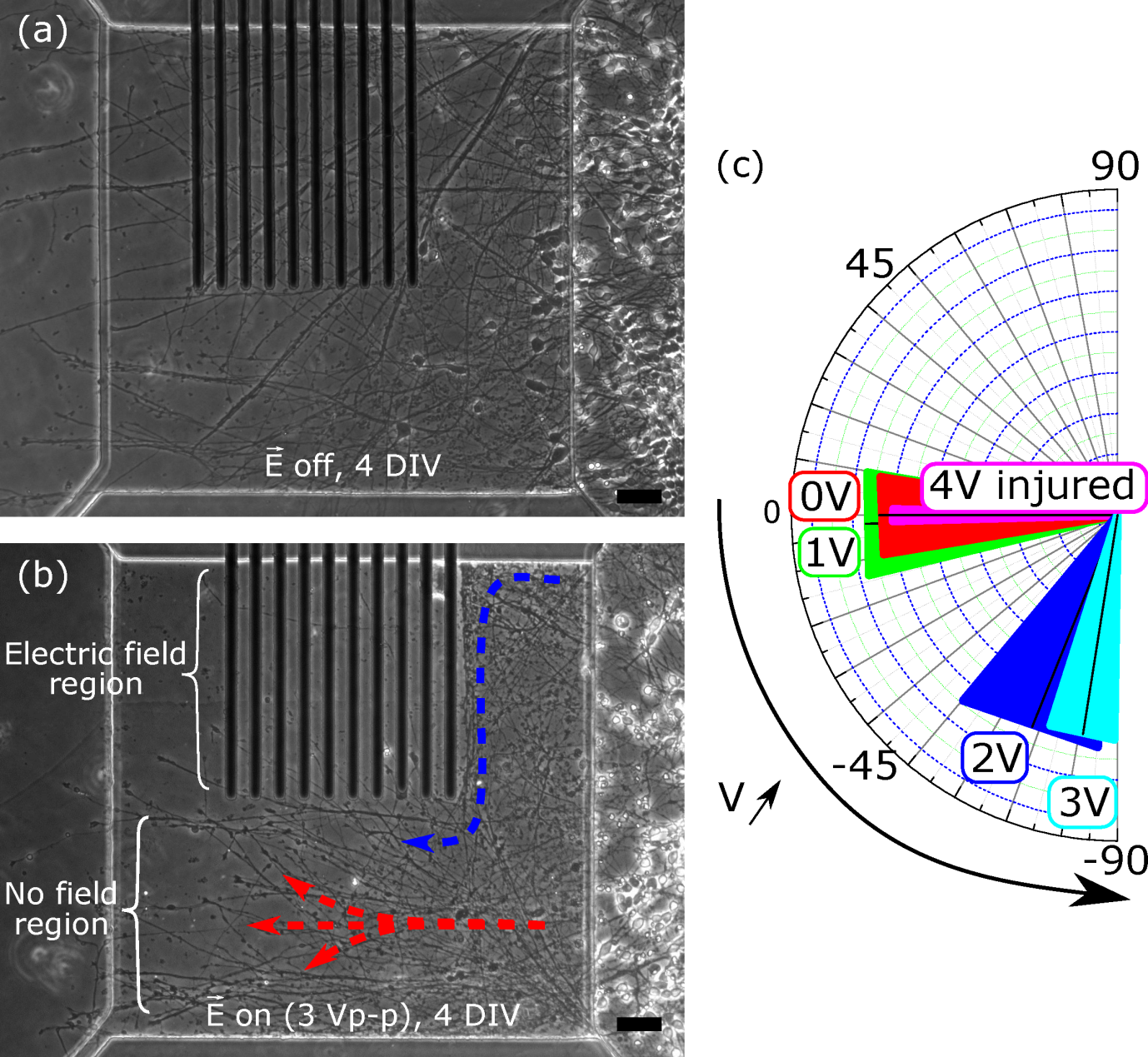


Figure S2: **Guiding neurites in a planar gels.** (a) Picture of the 5 μm high neurite microchannel wihout any field after 6 DIV. (b) Picture of the activated chip after 4 DIV (3 Vpp, 150 kHz). In the control region (red arrow), neurites showed approximately the same growth speed and density as in the control chip. In contrast however, the electric field region was nearly free from neurite growth. Neurites turned in front of the first electrode and avoided the electric field lines until reaching regions of lower field strength (blue arrow). (c) Plot of the change in growth direction evaluated as function of the applied voltage (n=3 chips). The black lines are the median angle for each voltage and the width of the cone shows standard deviation. In all experiments with a scaffold of 5 μm high, the presence or absence of scaffold in the channel resulted in the same behavior. All scale bars indicate 50 µm.


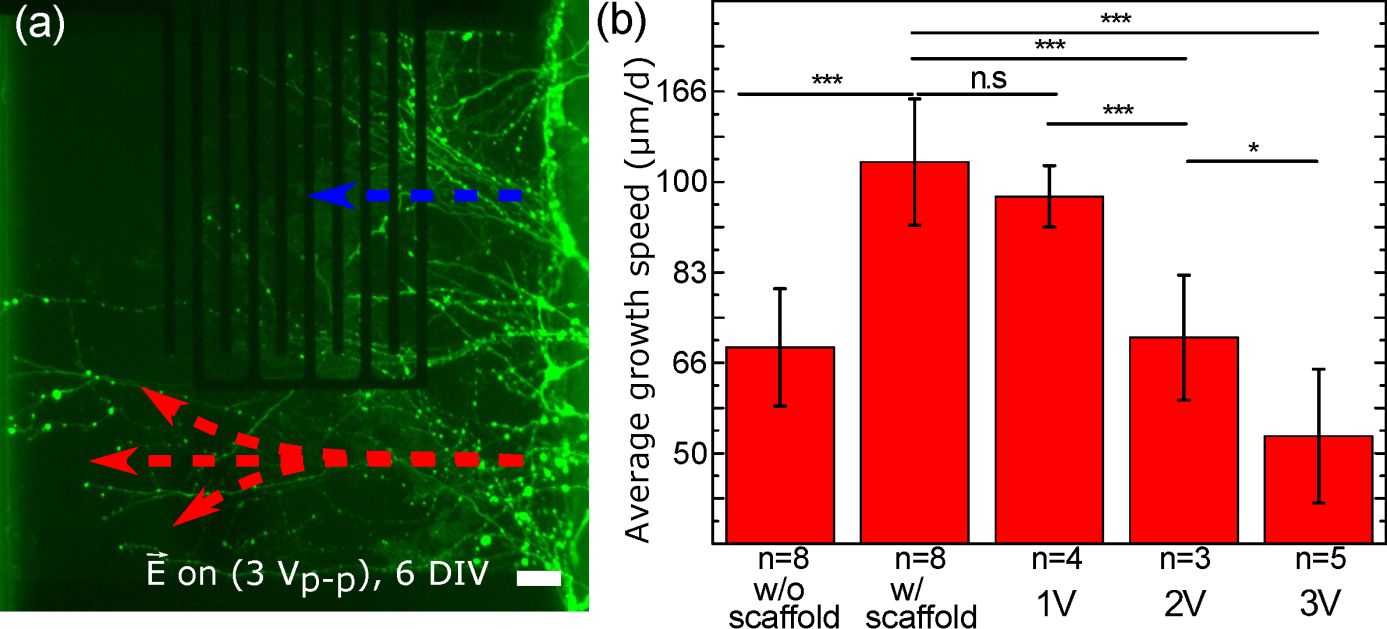


Figure S3: **AC fields can slow down neurites growth in collagen scaffolds.** (a) Fluorescence picture of neurites extending through a channel with a height of 10 μm with the electric field turned on after 6 DIV (3 Vpp, 150 kHz). Control chips with inactivated electrodes were used with and without scaffolds. (b) Average neurite growth speed after 6 DIV. The presence of a scaffold enhances the growth speed by ~ 30% over functionalized surfaces. Applying 2 Vpp and 3 Vpp significantly reduced the length of the neurites. A potential explanation for growth promotion is the alignment of collagen fibers parallel to the growth direction as a result of the scaffold filling protocol (35). The ”n” indicates the number of independent experiments repeats. Scale bar indicates 50 µm.


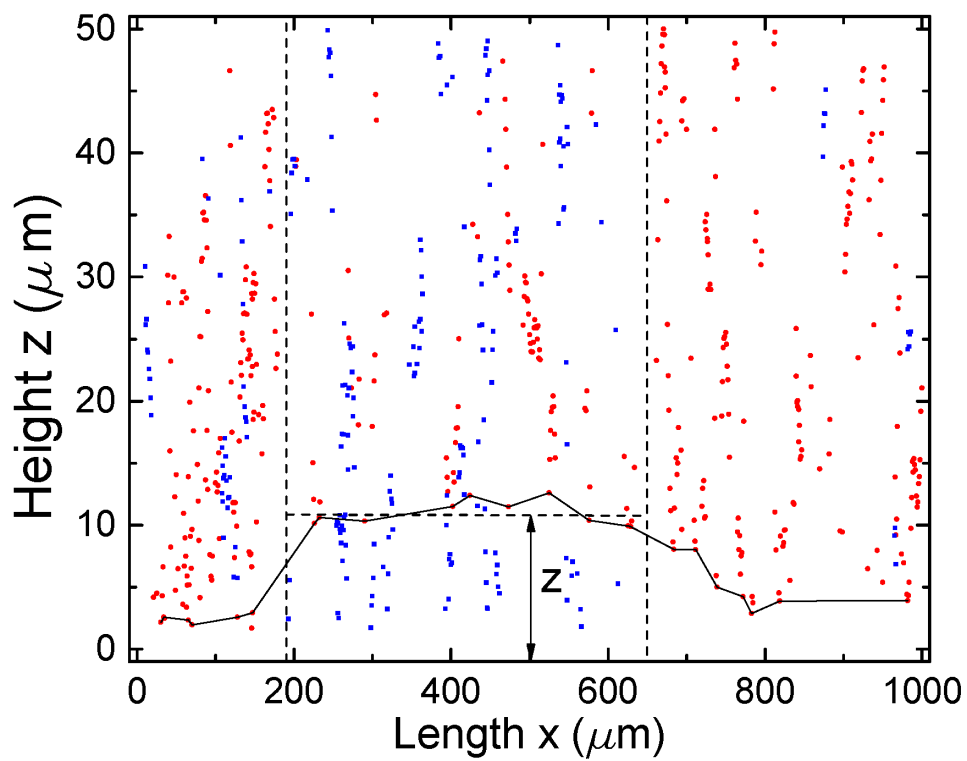


Figure S4: **Neurite growth in 3D gels.** Side view reconstruction of 3D images produced by the confocal microscopy for fluorescent neurites extending through a 50 μm high collagen-filled channel after 6 DIV without the field (blue points) and with the field (3 Vpp, 150 kHz) (red points). When applying the field, neurites growing close to the electrodes increased their location in z when over the electrodes (solid line). The lowest height of neurites in the field region is represented by the horizontal dashed line and quantified for several voltages (Fig. 2f). The vertical dashed lines show the spatial locations of the electrodes.

Movie S5: 3D confocal reconstruction of neurites in collagen after being pushed up from the bottom electrodes.


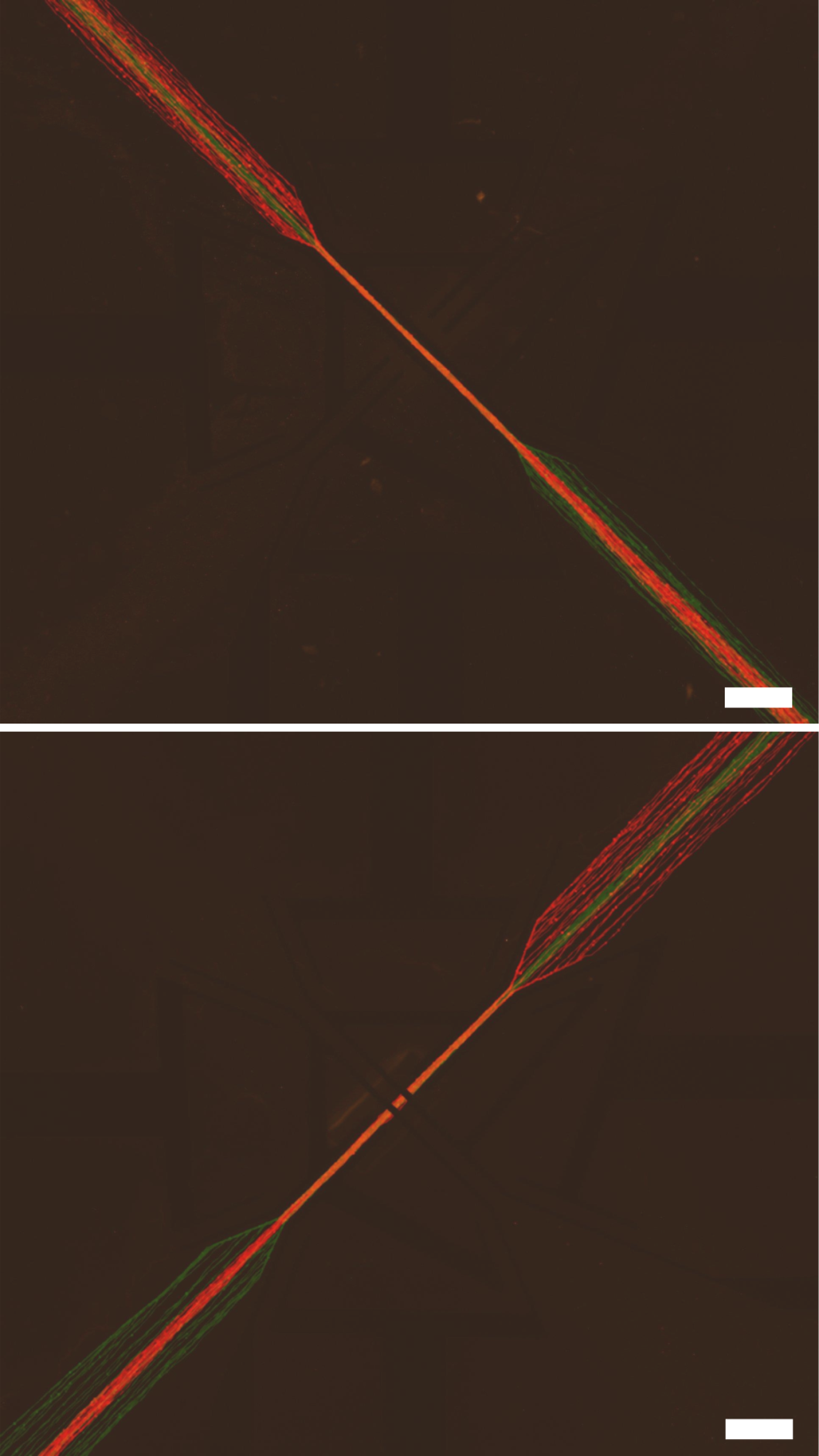


Figure S6: **Neurite bridges and bi-directionality of growth.** Fluorescence pictures of two facing populations of neurons bi-directionally connected in the neurite bridge device. The field was activated (150 kHz and 4 Vpp) and the HP was maintained low in the output reservoirs. Scale bars indicate 100 µm. The connectivity matrix were respectively for the upper and lower pictures:


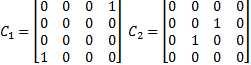


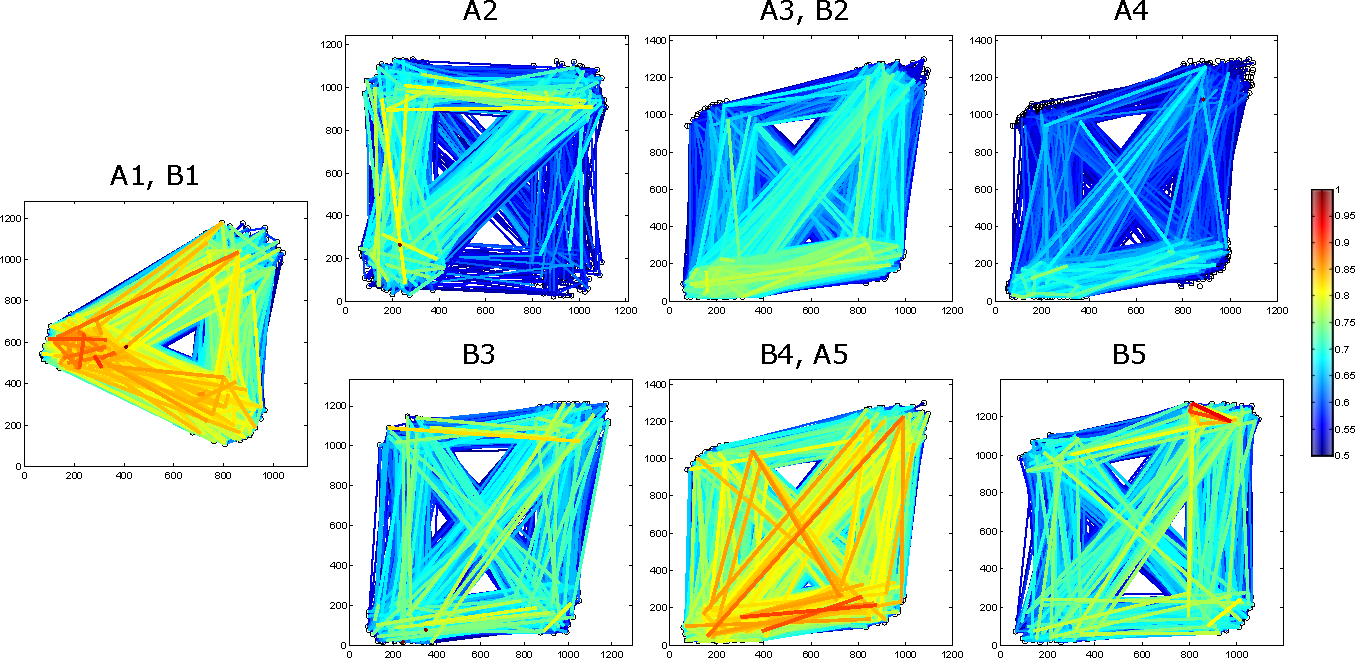


Figure S7: **Functional networks of basic brain motif**. Reconstruction of the functional networks of the basic brain motifs. The color coding indicates the strength of the correlation between each node according to the scale bar.

Table T1: **Degree distribution of basic motifs**

|  | A1,B1 | A2 | A3,B2 | A4 | B3 | B4,A5 | B5 |
| --- | --- | --- | --- | --- | --- | --- | --- |
| Clustering coefficient σ | 1.57±0.22 | 9.53±0.65 | 8.53±0.54 | 7.98±0.74 | 4.81±0.23 | 5.17 ± 0.28 | 5.24 ± 0. 42 |
| shortest path length λ | 0.91±0.07 | 1.46±0.06 | 1.37±0.05 | 1.32±0.07 | 1.07±0.03 | 1.08±0.03 | 1.07 ± 0.04 |
| small-world parameter σ/λ | 1.72±0.04 | 6.51±0.08 | 6.21±0.07 | 6.01±0.09 | 4.48±0.04 | 4.79±0.05 | 4.87 ± 0.09 |


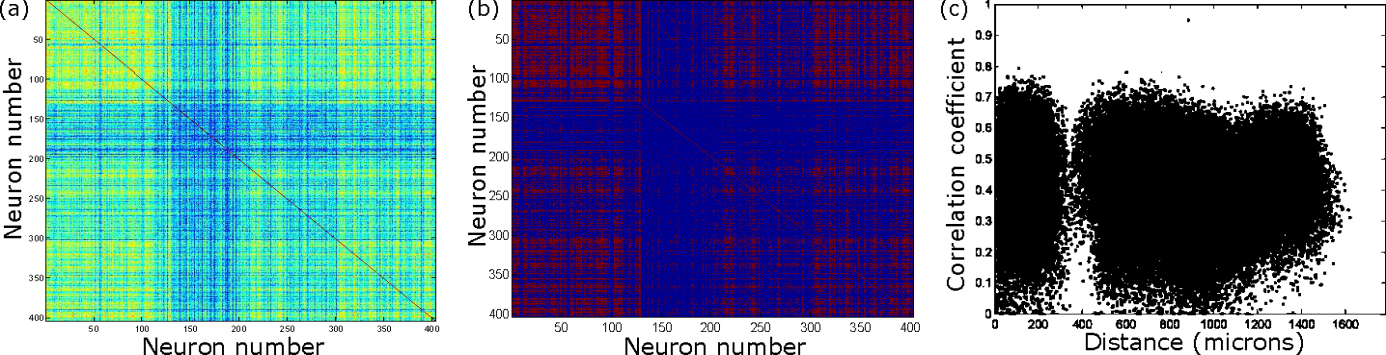


Figure S8: **Cross-correlation outputs for the B5 motif**. (a) Correlation with color strength coding and (b) adjacency matrix of the motif B5 using the cross-correlation between intra and inter-population spikes. (c) Plot of the correlation coefficients as a function of distance.

Supplementary note 1

**Neural networks of any complexity can be successfully design on a chip, theoretical proof.**

By combining the unit operations (diodes and bridges), we hypothesize that neural networks of any complexity can be successfully design on a chip. Hereafter is a theoretical proof of this hypothesis where the network is an actual biological neural network composed of nodes (reservoir of neurons bodies) and edges (connecting microchannels with neurites).

Suppose *N=(V,E)* represents a network with *n* nodes, where *V={v_1_,…,v_n_}* is the set of nodes and *E* is the set of edges. Adjacent nodes are connected by straight lines. A point *p* in the plane is called a *k*-intersection point (or an intersection point with degree *k*) if *k* edges pass through it. In the following, we show that any graph (network) can be drawn in the plane having at most 2-intersection points. To show it, we propose an iterative method to remove *k*-intersection points from the graph where *k>2*.

Suppose *p_1_* is a *k*-intersection point where *k>2*. Suppose an edge connecting nodes *v_i_* and *v_j_* is passing through *p_1_*. We claim that the position of node *v_j_* can be changed so that *p_1_* is reduced to a (*k-1*)-intersection point, while other intersection points remain the same. Suppose *v_j_* is connected to the nodes *N(v_j_)*, and suppose the graph has intersection points *P*. Denote *L* as the set of  all lines connecting points from the set *N(v_j_)* to the points in the set *P* . If we move *v_j_* to a point in the plane which is not in *L*, none of the other intersection points will change, while the degree of the intersection point *p* would be decreased to *k-1*. Since we have finite number of vertices, the set *L* has measure zero, and this procedure is always possible with probability one. We repeat this procedure for all intersection points with degree higher than 2 so that all intersection degrees are at most 2. This completes the proof.
